# Biomechanical Comparison of a New Memory Compression Alloy Plate versus Traditional Titanium Plate for Anterior Cervical Discectomy and Fusion: A Finite Element Analysis

**DOI:** 10.1155/2020/5769293

**Published:** 2020-07-17

**Authors:** Jiantao Liu, Runqing Wang, Hongbo Wang, Yanbiao Wang, Dongbo Lv, Pan Diao, Shihan Feng, Yanzheng Gao

**Affiliations:** ^1^Department of Spine and Spinal Cord Surgery, Henan Provincial People's Hospital, 450003, No. 7 the Weft Five Road, Zhengzhou, Henan, China; ^2^Department of Heart Center, First Hospital of Lanzhou University, 730000, No. 1 Dong Gang West Road, Lanzhou, Gansu., China; ^3^Department of Orthopedics, Second Affiliated Hospital of Xi'an Jiaotong University, 710004, No. 157 West Five Road, Xi'an, Shaanxi, China; ^4^Department of Medicine and Biosciences, Kansas City University, Kansas City, USA

## Abstract

**Objective:**

To compare the biomechanical properties of a new memory compression alloy plate and traditional titanium plate after anterior cervical discectomy and fusion (ACDF).

**Methods:**

A finite element model of the C3-7 segments was developed and validated. The C5-6 disc was removed, and an intervertebral cage made of peek material was implanted. Then, a new memory compression alloy plate composed of Ti-Ni memory alloy and a traditional titanium plate were integrated at the C5-6 segment. All models were subjected to a load of 73.6 N to simulate the head weight and 1 Nm of flexion-extension, lateral bending, and axial rotation. The range of segmental motion (ROM) and stress on the prostheses, adjacent discs, and endplates were analyzed.

**Results:**

Compared with intact status, ACDF with the new prothesis and traditional titanium plate reduced the ROM of C5-6 in six directions by 95.2%-100% and increased that of adjacent discs (C4-5 and C6-7) by 4.8%-112.5%. Adjacent disc stress peaks were higher for the traditional titanium plate (0.7-4.2 MPa) than for the new prosthesis (0.6-4.1 MPa). Endplate stress peaks were the highest in ACDF with the new prosthesis (15.6-53.3 MPa), followed by ACDF with traditional titanium plate (5.0-29.4 MPa). Stress peaks were significantly lower for the new prothesis (12.8-52.3 MPa) than for the traditional titanium plate (397.0-666.1 MPa).

**Conclusions:**

The new prosthesis improved the immediate stability of the surgical site and had an elastic modulus that was smaller than that of traditional titanium plate, making it conducive to reducing stress shielding and the impact on the adjacent intervertebral disc.

## 1. Introduction

Cervical spondylosis is a common degenerative disease that often causes pain in the neck, weakness in the lower limbs, and dizziness, nausea, and other discomfort symptoms [1–3]. Surgery is often required to relieve those symptoms when the disease progresses to a certain extent [4–6]. Anterior cervical discectomy and fusion (ACDF) was first reported by Bailey and Badgley [7], Smith and Robinson [8], and Colward [9] in the 1950s and early 1960s and is considered the gold standard treatment for single-level cervical degenerative disc disease causing radiculopathy or myelopathy [10–12]. In this operation, cages are widely used for interbody fusion in clinical practice. Despite the overwhelming superiority of this approach, patients treated with stand-alone cages are associated with many complications, such as graft subsidence, graft compression fractures, and graft dislocation [13–18]. To overcome these graft-related complications, the anterior cervical plates (ACPs) are fixed [19, 20]. A large number of clinical applications have shown that ACPs can enhance the stability of surgical sites, promote fusion, and reduce the subsidence of the cages [21, 22]. However, the use of a titanium ACP may be associated with complications including dysphagia and soft tissue injury because of its large volume [6, 14]. In addition, the high elastic modulus and rigid fixation of titanium ACP might result in graft stress shielding, leading to screws backing out and looseness of the titanium plate [23]. Thus, it is of great clinical significance to develop a new anterior cervical plate to solve these defects.

Ni-Ti alloy material is a shape memory alloy and has been widely used in orthopedic implants because of its advantages such as abrasion resistance, fatigue resistance, corrosion resistance, and good biocompatibility [24–26]. In view of this, we have developed a novel ACP with Ni-Ti memory alloy called a memory compression alloy plate. Since it was mainly invented by Professor Gao Yanzheng, we named the new prosthesis “GYZ plate” with his initials. This device has advantages, including its small size, convenient implantation, small elastic modulus, and continuous pressure. At present, this prosthesis has obtained an invention patent (Number: ZL200810141238.0). To test the stability and effects of stress on adjacent tissues and lay a biological foundation for its biological application, we performed a biomechanical assay to compare the new prosthesis to traditional titanium plate in a three-dimensional nonlinear finite element (FE) model.

## 2. Methods and Materials

### 2.1. Ethics Statements

This study was carried out in accordance with the Code of Ethics of the World Medical Association (Declaration of Helsinki) and approved by the Ethics Committee of Zhengzhou University (Number: 2019012). A computed tomography (CT) scan of the cervical spine was obtained with the informed consent of volunteers, and the dissemination of relevant data was allowed for academic exchange.

### 2.2. Finite Element Model of the Intact C3-7 Spine

According to the processes reported by Liu et al. [27], .CT images of C3-7 spine were obtained at 0.625 mm intervals from a young male volunteer (age: 20 years, height: 173 cm, weight: 70 kg) without radiographic changes in cervical vertebrae or a history of cervical disc disease. All images were saved in DICOM format and then imported into Mimics (Materialise Inc., Leuven, Belgium) to reconstruct the geometric structure of each vertebra. All vertebrae were saved in STL format and imported into Geomagic studio 12 (Geomagic Inc., North Carolina, USA) to smooth and reduce noise caused by the vertebral surface. The processed model was saved in IGES format and imported into Pro/Engineer5.0 (Parametric Technology Corporation, Massachusetts, USA) to generate solid models of each vertebra. The cortical shell, cancellous bone, annulus fibrosus, and nucleus pulposus, as well as other cartilage models were subsequently constructed. Mesh models of the bones, intervertebral discs, and ligaments were constructed using HyperMesh (Altair Engineering, Inc., Troy, Michigan, USA). Using Abaqus (Hibbitt, Karlsson and Sorenson, Inc., Providence, Rhode Island, USA), models were assembled, and FE analysis was performed.

**Figure 1:**
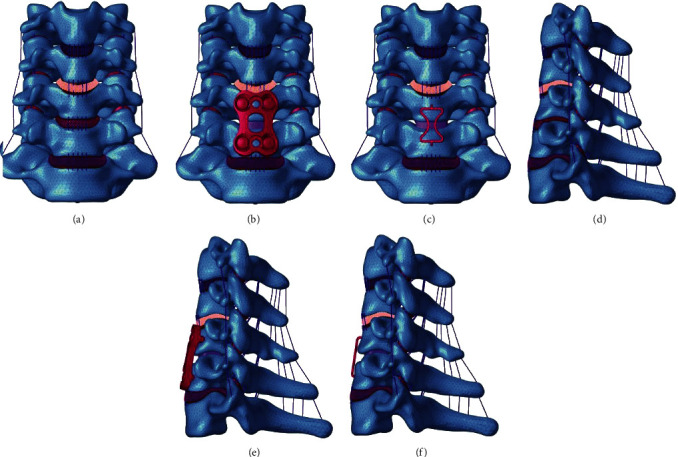
Finite element models of different groups: (a–c) front view of the three groups, (d–f) sagittal view of the three groups.

As shown in Figures 1(a) and 1(d), the FE model of the intact C3-7 cervical spine consisted of 5 vertebrae, 4 intervertebral discs, the anterior longitudinal ligament, the posterior longitudinal ligament, the capsular ligament, the interspinous ligament, and the ligamentum flavum. The thickness of the cortical shell was defined as 1.5 mm. The nucleus pulposus was modeled as an incompressible fluid, and its volume accounted for 30%-40% of the intervertebral disc. The ligaments were modeled as truss elements that responded nonlinearly only to tension. There were 632470 nodes and 296156 elements in the intact disc model, which was used to eliminate the effect of mesh on the accuracy of the calculations. All the element types and material properties of the intact FE models were defined based on previous publications [27, 28] (Table 1). To validate the intact C3-7 FE model, intervertebral (segmental) ranges of motion (ROMs) in response to 1.0 Nm loads were compared with outcomes described in previous publications [29, 30].

### 2.3. Finite Element Modeling of ACDF with Different Implants

Figures 1(b) and 1(e) show 3D model diagrams of successful ACDF modeled with traditional titanium plate, while Figures 1(c) and 1(f) show 3D model diagrams of successful ACDF modeled with the new prosthesis. The modeling process was performed as follows. The discectomy was simulated by removing the C5-6 intervertebral disc and the corresponding anterior and posterior longitudinal ligaments. After decompression, a suitably sized polyetheretherketone interbody cage (7 × 12 × 12 mm) (Compact CORNERSTONE-SR [Medtronic Inc., Memphis, Tennessee, USA]) was modeled and inserted into the intervertebral space to simulate interbody fusion. Both ends of the cage were verified to be in complete contact with the corresponding end plates. In the traditional titanium plate group, an anterior plate-screw system (traditional titanium plate and four screws) was placed at C5-6 to further stabilize the surgical segment. The length and width of the plate were 26 and 16 mm, respectively. The length and diameter of the screw were 16 and 4 mm, respectively. In the new prosthesis group, an integrated GYZ plate (the height, length, and width of the pressurized part of the GYZ plate were 20, 13.5, and 14.3 mm, respectively) made of Ni-Ti memory alloy was placed in the same position that a traditional titanium plate would be placed in. The element types and material properties of different implants were also defined based on previous publications [31] (Table 2). For all surgical models, the interfaces at the cage-end plate and screw-bone were defined as a tied contact conditions to simulate a complete fusion status.

### 2.4. Loading and Boundary Conditions

For all FE models, all freedoms of the inferior surface of the C7 vertebra were fixed, and 73.6 N of vertical downforce load was applied to the superior surface of the C3 vertebra to simulate the head weight. At the same time, moment loads of 1.0 Nm were also applied to the superior surface of the C3 vertebra to produce flexion, extension, left and right bending, and rotation. The ROM and stress on the prostheses, adjacent discs, endplates, and facet joints of FE models induced in different groups in response to the maximum load (1 Nm) were analyzed.

## 3. Results

### 3.1. Model Validation

**Figure 2:**
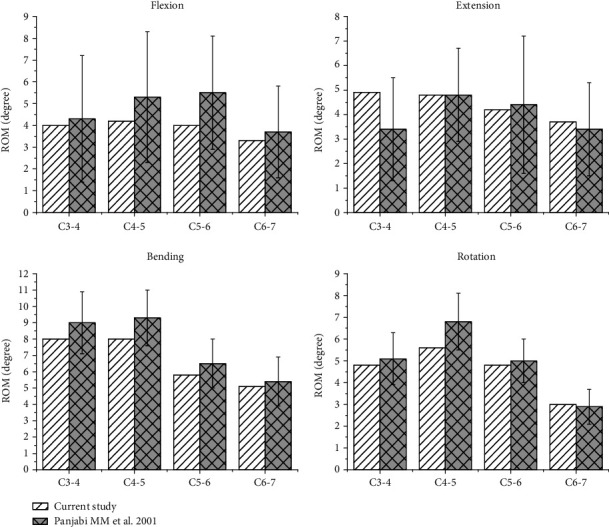
Comparison between the predicted C3-7 ROMs (under moments of 1 Nm) and those obtained in Panjabi's previous biomechanical studies. The ROMs for bending refer to the sum of two directions, left and right lateral bending, and the ROMs for rotation.

Figure 2 shows the comparison between the predicted C3-7 ROMs (under moments of 1 Nm) and those obtained in Panjabi's previous biomechanical studies [30], which was reported by Liu et al. [27]. The intersegmental ROMs of the intact cervical FE model at C3-4, C4-5, C5-6, and C6-7 were 4.0, 4.2, 4.0, and 3.3 degrees, respectively, during flexion; 4.9, 4.8, 4.2, 3.7 degrees, respectively, during extension; 8.0, 8.0, 5.8, 5.1 degrees, respectively, during left and right bending; and 4.8, 5.2, 4.8, 3.0 degrees, respectively, during left and right rotation. All the intersegmental ROMs of the intact FE model fell within one standard deviation of Panjabi's experimental data, suggesting that the intact C3-7 FE model used in the current study was successfully constructed and could be used for further analysis.

### 3.2. ROM in the Intact and Implanted Spine Models

**Figure 3:**
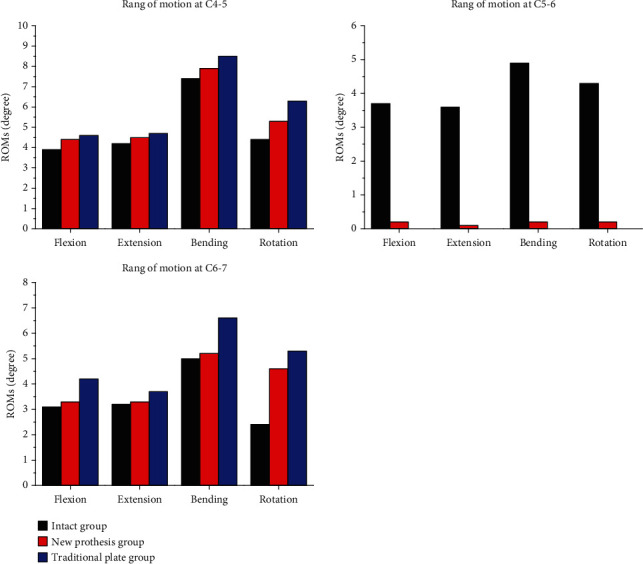
Comparisons of the ranges of motion observed at different discs among the three groups.

As shown in Figure 3 and Table 3, the ROMs obtained for the intact model during flexion, extension, bending, and rotation were 3.9, 4.2, 7.4, and 4.4 degrees at the C4-5 disc; 3.7, 3.6, 4.9 and 4.3 degrees at the C5-6 disc; and 3.1, 3.2, 5.0, and 2.4 degrees at the C6-7 disc, respectively. Postoperatively, the ROMs of ACDF performed using a traditional plate during flexion, extension, bending, and rotation were significantly higher, at 4.6~8.5 degrees, at the C4-5 disc; and 4.2~6.3 degrees, at the C6-7 disc. However, the ROMs of ACDF performed using a traditional plate in the four directions above the C5-6 disc were significantly lower, at zero. The ROMs of ACDF performed using a new prosthesis, a GYZ plate, showed a trend similar to that of the traditional plate group, including lower ROMs at the fusion disc (C5-6) and higher ROMs at the adjacent discs (C4-5, C6-7). The ROMs of the C5-6 disc were 0.1~0.2 degrees during flexion, extension, bending, and rotation, respectively; while the corresponding ROMs were 4.4~7.9 degrees, at the C4-5 disc and 3.3~5.2 degrees, at the C6-7 disc.

### 3.3. Intervertebral Disc Stresses

**Figure 4:**
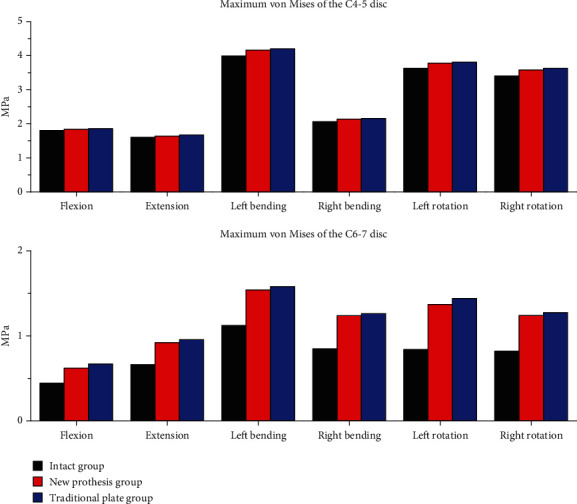
The maximum von Mises stresses in the adjacent discs (C4-5 and C6-7) in the three groups.

Figure 4 shows the maximum von Mises stresses recorded in the adjacent discs (C4-5 and C6-7) in the three groups. The disc stress peaks were the highest in the construct in which ACDF was performed using a traditional plate; in this group, the flexion, extension, left bending, right bending, left rotation, and right rotation stresses were 1.86, 1.61, 4.21, 2.16, 3.82, and 3.63 MPa at C4-5, and 0.67, 0.96, 1.58, 1.26, 1.44, and 1.27 MPa at C6-7, respectively. During the same types of movement, the disc stress peaks were slightly lower when the new prosthesis was used for reconstruction in ACDF, at 1.84, 1.64, 4.17, 2.14, 3.78, and 3.58 MPa, respectively, at C4-5 and 0.62, 0.92, 1.54, 1.24, 1.37, and 1.24 MPa, respectively, at C6-7. The disc stress peaks were the lowest in the intact group, at 1.81, 1.60, 3.99, 2.06, 3.63, and 3.41 MPa, respectively, at C4-5 and 0.45, 0.66, 1.12, 0.85, 0.84, and 0.82 MPa, respectively, at C6-7.

### 3.4. Cortical End Plate Stresses

**Figure 5:**
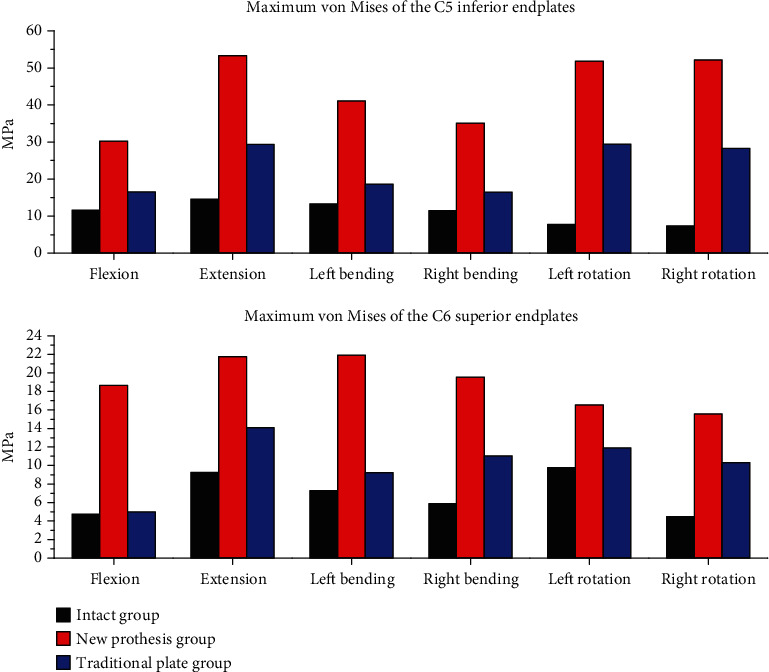
The maximum von Mises stresses at the C5 inferior endplates and C6 superior endplates in the three groups.

**Figure 6:**
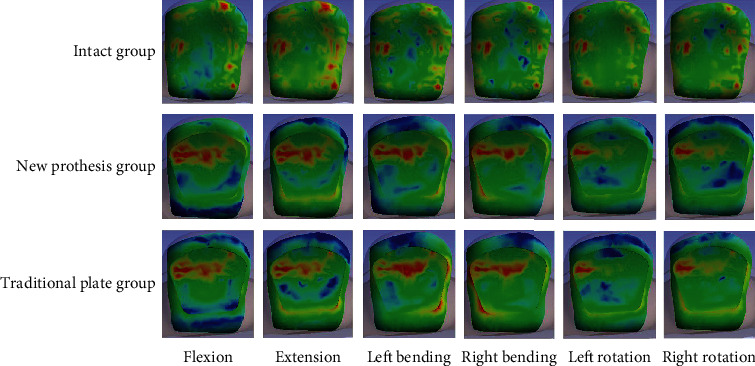
The distributions of stresses in the C5 inferior endplates in the three groups during flexion, extension, left bending, right bending, left rotation, and right rotation.

Figure 5 and Table 4 show the maximum von Mises stresses recorded at the endplates. The stress peaks of the endplates were highest for ACDF with a new prosthesis, followed by the traditional plate group. The stress peaks of the endplates were lowest in the intact group. The maximum von Mises stresses in these three groups were 30.23, 16.58, and 11.64 MPa, respectively, at the C5 inferior endplate and 18.67, 5.01, and 4.76 MPa, respectively, at the C6 superior endplate during flexion; 53.33, 29.39, and 14.57 MPa, respectively, at the C5 inferior endplate and 21.76, 14.08, and 9.28 MPa, respectively, at the C6 superior endplate during extension; 41.10, 18.67, and 13.30 MPa, respectively, at the C5 inferior endplate and 21.91, 9.25, and 7.28 MPa, respectively, at the C6 superior endplate during left bending; 35.12, 16.50, and 11.47 MPa, respectively, at the C5 inferior endplate and 19.54, 11.02, and 5.88 MPa, respectively, at the C6 superior endplate during right bending; 51.83, 29.43, and 7.81 MPa, respectively, at the C5 inferior endplate and 16.55, 11.90, and 9.76 MPa, respectively, at the C6 superior endplate during left rotation; and 52.13, 28.31, and 7.41 MPa, respectively, at the C5 inferior endplate and 15.57, 10.31, and 4.48 MPa, respectively, at the C6 superior endplate during right rotation.  Figure 6 shows the distributions of stresses in the C5 inferior endplates.

### 3.5. GYZ and Traditional Plate Stresses

**Figure 7:**
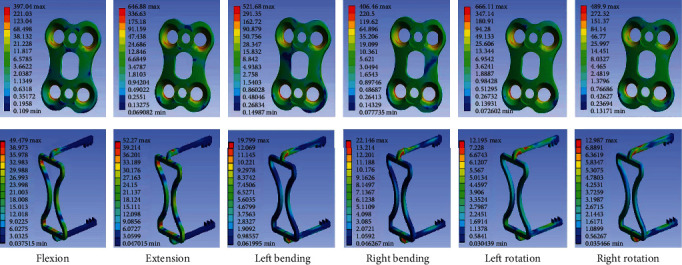
The distributions of stresses in the traditional plate and new prosthesis groups during flexion, extension, left bending, right bending, left rotation, and right rotation.

Figure 7 shows the distributions of stresses in the traditional plate and new prosthesis groups. According to the results, the maximum von Mises stresses observed for the traditional plate during flexion, extension, left bending, right bending, left rotation, and right rotation were 397.04, 646.88, 521.68, 406.46, 666.11, and 489.90 MPa, respectively. However, the maximum von Mises stresses observed for the new prosthesis were significantly lower, at 49.48, 52.27, 19.80, 22.15, 12.20, and 12.99 MPa, respectively.

## 4. Discussion

With changes in modern people's way of life and work, the incidence of cervical spondylosis is increasing year by year. Anterior cervical discectomy and fusion (ACDF) has been the gold standard treatment for these diseases for the past 60 years when conservative treatment fails [9]. ACPs are often used to ensure the stability of the fusion site. However, some studies have shown that rigid fixation of ACPs can increase the mobility and stress of adjacent intervertebral discs, leading to the accelerated degeneration of adjacent intervertebral discs. In addition, large ACPs are also prone to postoperative dysphagia and soft tissue injury. Therefore, the development of a small ACP that can reconstruct the stability of the surgical site and minimize the impact on the adjacent intervertebral discs would be of great clinical significance. To solve the above problems, our group developed a new ACP made of Ni-Ti memory alloy; this plate not only performed a memory pressure function but also has a small volume and is convenient to implant. We conducted this study to evaluate the biomechanical properties of the new prosthesis and its effects on adjacent intervertebral spaces after implantation.

In this study, a three-dimensional FE model of the lower cervical spine was developed and validated using published experimental data. ROM was evaluated in an intact model and models with prostheses. The stresses exerted on the prostheses and adjacent discs were analyzed.

According to our results, the ROMs at the surgical site were significantly lower during flexion, extension, bending, and rotation in both the traditional plate group and the new prosthetic group than in the intact group, consistent with the reduced mobility observed at the surgical site after fusion. It was also further confirmed that these two types of ACPs reconstructed the immediate stability of the surgical site. However, the ROMs of adjacent discs (C4-5 and C6-7) were larger in the groups that underwent surgery with different protheses than in the intact group, indicating that both types of ACPs affected the activity of adjacent intervertebral discs after implantation. As the ROMs of adjacent intervertebral discs were smaller in the new prosthesis group than in the traditional plate group, the stress exerted on the adjacent intervertebral disc was lower than that exerted on the traditional titanium plate group, consistent with the distribution of stress in the adjacent disc. Thus, the effect on adjacent intervertebral discs was weaker in the new prosthesis group than in the traditional plate group. Compared with traditional titanium plates, the new plate slowed down the degeneration of the adjacent discs to some extent.

The results obtained for the maximum von Mises stresses of the endplates showed that the stress peaks of the endplates were lowest in the intact group. The reason may be related to the elastic cushioning effect of the intervertebral disc, which reduces the stresses on the adjacent endplates to some extent. The stress peaks of the endplates were higher in both ACDF groups that used different prostheses than in the intact group, indicating that the current intervertebral fusion cage belongs to the group of rigid prostheses and did not simulate the elastic cushioning function of intervertebral discs. Therefore, the stress distribution of adjacent endplates would increase after implantation. The stress peaks of the endplates were higher in the new prothesis group than in the traditional plate group. This indicated that the fixed stiffness was higher for the traditional titanium plate than the new prosthesis, which dispersed the stress applied to the endplates and cage to some extent. However, some studies have shown that stress stimulation is good for bone growth. Therefore, stronger fixation is not conducive to the bony fusion between the intervertebral fusion cage and the adjacent endplates. Therefore, from this point of view, the new prosthesis was more conducive to facilitating bone fusion between the interbody fusion cage and the adjacent endplate. In addition, the new prosthesis is made of memory alloy and can be restored to its original shape at 37°C. The new prosthesis was stretched and deformed at 0°C and then placed into the body. Using the warming effect of human body temperature and gradually rewarm the new prosthesis to 37°C. In the process of reheating, the new prosthesis gradually returns to its original shape, allowing continuous pressure to be applied to the surgical area while promoting bone fusion at the surgical site.

According to the results obtained for maximum stress distribution of the new prosthesis and the traditional titanium plate, the level of stress on the traditional titanium plate was higher, further verifying that fixation stiffness was higher for the traditional titanium plate than for the new prosthesis. Strong stiffness increases the elastic modulus, leading to local stress shielding and thereby causing adjacent disc degeneration, prosthesis loosening, fracture, and other complications. However, the new prosthesis was effective in reducing local stress, and its elastic modulus was closer to that of bone.

This study has several limitations. In this study, FE modeling of the intact cervical spine was based on CT data obtained in a young and healthy man, and these data might have neglected the influence of degenerative pathologies on the biomechanical performance of the cervical spine. In addition, the results obtained for the ACDF procedures were based on a single FE model. The biomechanical characteristics of this FE model may not be analogous to the situation experienced by the cervical spine during severe pathologic changes, such as severe osteoporosis and cervical kyphosis, which significantly decrease the mechanical strength of the vertebral body and change the kinematics of the involved level, respectively. Further studies are needed to analyze these specific situations.

In conclusion, this study shows that this new prosthesis not only improved the immediate stability of the surgical site but also had an elastic modulus that was smaller than that of traditional titanium plate, making it conducive to reducing stress shielding and the impact on the adjacent intervertebral disc. In addition, this new type of prosthesis is integrated into one body that is not only small in size and convenient to implant, but it can also reduce postoperative soft tissue injury, maintain pressure to promote bone fusion of the adjacent endplate and intervertebral fusion cage, and reduce postoperative screws. Therefore, the new prosthesis is expected to be further popularized in clinical practice to treat cervical spondylosis.

## Figures and Tables

**Table 1:** Material properties assigned to the intact FE model.

Component element	Type	Young modulus (MPa)	Poisson ratio	Cross-sectional area (mm^2^)
Cortical bone	C3D4	12000	0.3	/
Cancellous bone	C3D4	450	0.2	/
Endplate	C3D4	500	0.4	/
Articular cartilage	C3D4	10.4	0.4	/
Nucleus pulpous	C3D8H	1	0.49	/
Annulus fibers	T3D2	110	0.3	/
Annulus ground substance	C3D8H	3.4	0.4	/
Anterior longitudinal ligament	T3D2	10	0.3	6
Posterior longitudinal ligament	T3D2	10	0.3	5
Ligamentum flavum ligament	T3D2	1.5	0.3	5
Interspinous ligament ligament	T3D2	1.5	0.3	10
Supraspinous ligament	T3D2	1.5	0.3	5
Ligamenta intertransversaria	T3D2	1.5	0.3	5
Capsular	T3D2	10	0.3	46

**Table 2:** Material properties assigned to different prostheses.

Component element	Type	Young modulus (MPa)	Poisson ratio	Cross-sectional area (mm^2^)
PEEK	C3D4	3760	0.3778	/
Titanium alloy	C3D4	110000	0.3	/
Ti-Ni memory alloy	C3D4	65840	0.33	/

Note: PEEK refers to polyetheretherketone.

**Table 3:** The ranges of motion at different discs among the three groups (degrees).

Group		Flexion	Extension	Bending	Rotation
C4-5 disc	Group1	3.9	4.2	7.4	4.4
	Group2	4.6	4.7	8.5	6.3
	Group3	4.4	4.5	7.9	5.3
C5-6 disc	Group1	3.7	3.6	4.9	4.3
	Group2	0	0	0	0
	Group3	0.2	0.1	0.2	0.2
C6-7 disc	Group1	3.1	3.2	5.0	2.4
	Group2	4.2	3.7	6.3	5.3
	Group3	3.3	3.3	5.2	4.6

Note: Group1 refers to the intact group, Group2 refers to the traditional plate group, and Group3 refers to the new prosthesis.

**Table 4:** The maximum von Mises stresses at the adjacent endplates in the three groups (MPa).

Group		Flexion	Extension	Left bending	Right bending	Left rotation	Right rotation
C5 inferior endplate	Group1	11.64	14.57	13.30	11.47	9.76	7.41
	Group2	16.58	29.39	18.67	16.50	11.90	28.31
	Group3	30.23	53.33	41.10	35.12	16.55	52.13
C6 superior endplate	Group1	4.76	9.28	7.28	5.88	7.81	4.48
	Group2	5.01	14.08	9.25	11.02	29.43	10.31
	Group3	18.67	21.76	21.91	19.54	51.83	15.57

Note: Group1 refers to the intact group, Group2 refers to the traditional plate group, and Group3 refers to the new prosthesis.

## Data Availability

The data (saved in excel format) used to support the findings of this study are included within the supplementary information file(s).

## Supplementary Materials

Supplementary Materials (1) Animation of the new prosthesis implantation procedure. (2) The raw data on the results of the study.
